# Effect of perchlorate and thiocyanate exposure on thyroid function of pregnant women from South-West England: a cohort study

**DOI:** 10.1186/s13044-018-0053-x

**Published:** 2018-07-06

**Authors:** Bridget A. Knight, Beverley M. Shields, Xuemei He, Elizabeth N. Pearce, Lewis E. Braverman, Rachel Sturley, Bijay Vaidya

**Affiliations:** 1 NIHR Exeter Clinical Research Facility, Royal Devon & Exeter Hospital, University of Exeter Medical School, University of Exeter, Exeter, EX2 5DW UK; 2 Research & Development Department, Royal Devon and Exeter Hospital NHS Foundation Trust, Exeter, UK; 3 Section of Endocrinology, Diabetes & Nutrition, Boston University School of Medicine, Boston, USA; 4 Centre for Women’s Health, Royal Devon and Exeter Hospital NHS Foundation Trust, Exeter, UK; 5 Department of Endocrinology, Royal Devon and Exeter Hospital NHS Foundation Trust, Exeter, UK; 6 University of Exeter Medical School, Exeter, UK

**Keywords:** Perchlorate, Thiocyanate, Iodine, Thyroid, Pregnancy, Birth weight

## Abstract

**Background:**

Iodine is important for thyroid hormone synthesis, and iodine deficiency in pregnancy may impair fetal neurological development. As perchlorate and thiocyanate inhibit sodium-iodide symporter reducing the transport of iodine from circulation into the thyroid follicular cells, environmental exposure to these substances in pregnancy may impair maternal thyroid hormone synthesis. We aimed to explore the impact of perchlorate and thiocyanate exposure on thyroid status in a cohort of pregnant mothers from South West England.

**Methods:**

Urine samples were obtained from 308 women participating in a study of breech presentation in late pregnancy. They had no known thyroid disease and a singleton pregnancy at 36–38 weeks gestation. Samples were analysed for urinary concentrations of iodine (UIC), perchlorate (UPC) and thiocyanate (UTC). Blood samples were taken for free T4 (FT4), thyrotropin (TSH) and thyroid peroxidase antibodies (TPO-Ab). Baseline data included age, parity, smoking status, ethnicity and BMI at booking. Following delivery, data on offspring’s sex, gestational age at birth and birthweight were collected.

**Results:**

Participants had a mean (SD) age 31 (5) years, median (IQR) BMI 24.4 (22.0, 28.3) kg/m^2^, 42% were primiparous, 10% were smokers, and 96% were Caucasian. Median UIC was 88 μg/l, and 174/308 (57%) women had UIC < 100 μg/l. Log transformed UPC negatively correlated with FT4, but not with TSH, in the whole cohort (*r* = − 0.12, *p* = 0.03) and in the subgroup of women with UIC < 100 μg/l (*r* = − 0.15, *p* = 0.04). Regression analysis with the potential confounders (TPO-Ab status, UIC and UTC) identified UPC to be negatively associated with FT4 (*p* = 0.01). There was no correlation between UTC and FT4 or TSH. Maternal UPC or UTC was not associated with offspring birthweight.

**Conclusion:**

Environmental perchlorate exposure is negatively associated with circulating FT4 levels in third trimester pregnant women. This may have an adverse impact on neurocognitive development of the fetus.

## Background

Optimal level of maternal thyroid hormone in pregnancy is important for fetal neurological development, and as iodine is an essential component of thyroid hormone, iodine deficiency in pregnancy may impair fetal neurological development [1]. In recent years, there has been increasing concern that exposure to environmental pollutants during pregnancy may result in reduced maternal thyroid hormone synthesis affecting fetal neurodevelopment [2, 3].

Perchlorate and thiocyanate are common environmental pollutants that can disrupt normal thyroid function by reducing the uptake of iodine from the circulation into the thyroid follicular cells through competitive inhibition at the sodium-iodide symporter [2]. Perchlorate can be found in water, milk and some food crops, including green leafy vegetables and fruits. It was found to be present in 77 out of 342 food items tested in the UK between 2014 and 15 [4]. It is also present in fertilisers, rocket fuels, explosives, fireworks, road flares and air bags. Cigarette smoke is the main source of thiocyanate, but it can also be found in some vegetables of the Cruciferae family, such as cabbage, broccoli and cauliflower. Perchlorate is about 15 times stronger than thiocyanate as an inhibitor of the sodium-iodide symporter [5], and in pharmacological doses is known to suppress thyroid hormone synthesis [2]. However, there is a concern that even a low-level exposure of perchlorate or thiocyanate could disrupt thyroid hormone synthesis, particularly in the presence of iodine deficiency. This could lead to health hazards that may be more pronounced in vulnerable populations, such as pregnant women. Several studies have examined the effect of perchlorate and thiocyanate exposure during pregnancy on maternal thyroid function, however these showed conflicting results, and the potential adverse impact of exposure remains controversial [6–12].

Therefore, we aimed to study the impact of perchlorate and thiocyanate exposure on thyroid function of pregnant mothers from South West England where iodine deficiency has been shown to be common [13].

## Methods

### Participants

We studied 308 women participating in a study of breech presentation in late pregnancy [14]. They were recruited during routine antenatal care at the Royal Devon and Exeter Hospital (RD&E), a secondary care hospital in South-West England serving a population of about 450,000. They had a singleton pregnancy at 36–38 weeks gestation. They had no known thyroid disease, and were not on any drugs which may affect thyroid function. We collected baseline clinical data (including age, parity, smoking status, ethnicity and body mass index (BMI) at booking), and blood and spot urine samples from the participants. Following delivery, we collected data on sex, gestational age at birth and birth-weight of the babies; these data were unavailable for one baby as their mother moved away out of area before delivery.

### Sample analysis

**Blood samples** were analysed at the RD&E Blood Sciences department for serum thyroid stimulating hormone (TSH), free thyroxine (FT4) and thyroid peroxidase antibodies (TPO-Ab) using the electrochemiluminescent immunoassay, run on the Modular E170 Analyser (Roche, Burgess Hill, UK). Intra-assay coefficients of variations were < 5.3% for both TSH and FT4. The manufacturer’s population reference ranges were: TSH 0.35–4.5 mIU/l and FT4 11–24 pmol/l. Serum TPO-Ab titre above 34 IU/l was considered positive.

**Urine samples** were analysed for iodine, perchlorate, and thiocyanate concentration using ion chromatography-mass spectrometry at the Iodine Research Laboratory (Boston University, Boston, Massachusetts) [15]. The inter-assay coefficient of variation ranges from 3.1–8.2% for the three analytes.

### Statistical analysis

We assessed variables for normal distribution and log transformed (log) where appropriate. Where this did not result in a normal distribution, these data are presented as median (IQR). Correlations between two variables were assessed and results presented as Pearson correlation coefficients. Regression analysis was undertaken to identify independent predictors of thyroid status. As iodine deficiency may aggravate the effect of perchlorate and thiocyanate exposure, we also carried out subgroup analyses in women with urinary iodine concentration (UIC) less than 100 μg/l.

We analysed correlations between offspring birthweight (corrected for sex and gestational age at birth) and maternal urinary perchlorate concentration (UPC) and urinary thiocyanate concentration (UTC). We also carried out subgroup analyses considering birthweight (corrected for gestational age) of male and female infants separately.

With a sample size of 300, we can detect correlation coefficients *r* ≥ 0.12 as statistically significant at *p* < 0.05.

Statistical analysis was undertaken using SPSS (version 22).

## Results

### Baseline characteristics

Baseline clinical characteristics and thyroid function of the 308 participants are shown in Table 1. Ninety-six percent were Caucasian. The median UIC was 88 μg/l [13]. Fifty-seven percent (174 out of 308) women had UIC below 100 μg/l. The median UPC was 2.1 μg/l (IQR 1.4–3.8, range 0.3–143), and the median UTC was 436 μg/l (IQR 302–683, range 29–3290).

**Table 1:** Baseline Characteristics for total cohort (*n* = 308)

Characteristics	Figures
Age (years)^a^	31*(*5)
Booking BMI (kg/m^2^)^b^	24.4 (22.0, 28.3)
Gestation (weeks)^b^	37.0 (36.1, 37.6)
Primiparous	128 (42%)
Smoking	31 (10%)
Caucasian	295 (96%)
TSH (mIU/l)^b^	1.9 (1.4, 2.6)
Free T4 (pmol/l)^a^	12.0 (1.6)
TPO-Ab positive	16/305 (5%)
Urinary Iodine (μg/l)^b^	88 (55, 157)
Urinary Perchlorate (μg/l)^b^	2.1 (1.4,3.8)
Urinary Thiocyanate (μg/l)^b^	436 (302,683)

Data presented as ^a^mean (SD), ^b^ median (IQR) or (n) %

### Correlation between urinary perchlorate and maternal thyroid status

**Fig. 1:**
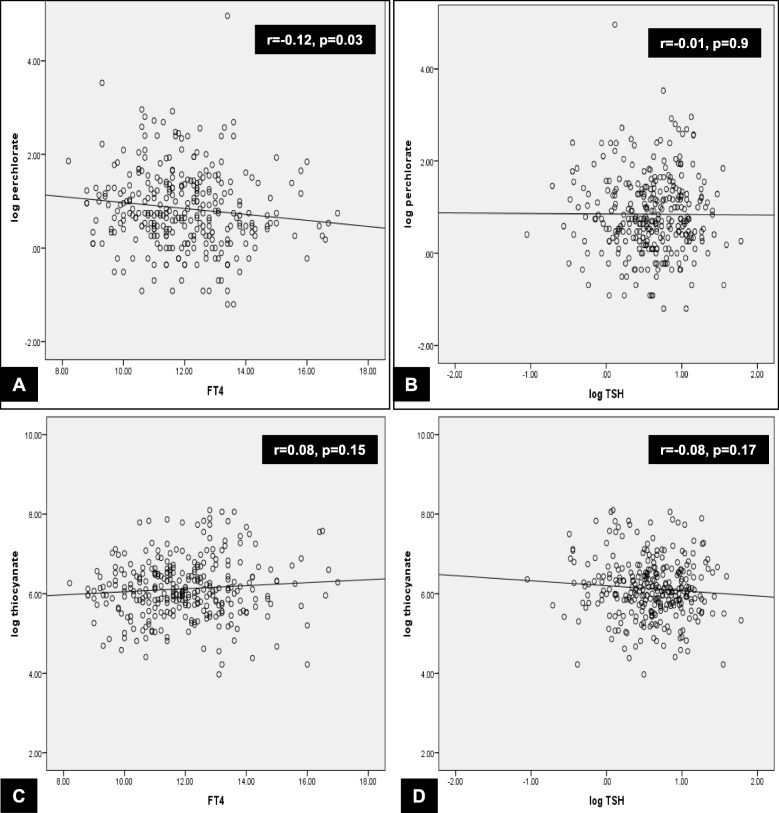
Correlations between log urinary perchlorate (Panels **a** and **b**) and thiocyanate (Panels **c** and **d**) with thyroid status

Log UPC was negatively correlated with FT4 (*r* = − 0.12, *p* = 0.03) but not with log TSH (*r* = − 0.01, *p* = 0.9) (Fig. 1, Panels a and b). In the subgroup of women with UIC less than 100 μg/l, log UPC remained negatively correlated with FT4 (*r* = − 0.15, *p* = 0.04) but not with log TSH.

### Correlation between urinary thiocyanate and maternal thyroid status

There was no correlation between log UTC and FT4 (*r* = 0.08, *p* = 0.15) or log TSH (*r* = − 0.08, *p* = 0.17) (Fig. 1, Panels c and d). Likewise, in the subgroup of women with UIC less than 100 μg/l, there was no correlation between log UTC and FT4 (r = 0.08, p = 0.17) or log TSH (r = − 0.08, p = 0.17).

### Multivariate analysis to assess the impact of potential confounders

We carried out multiple regression analysis to assess the impact of potential confounders (TPO-Ab, UIC, UPC and UTC). Maternal smoking was not included in the regression analysis because of its collinearity with thiocyanate levels (significant correlation between maternal smoking and log UTC, *r* = 0.4, *p* < 0.001). The regression analysis showed that only log UPC was significantly independently associated with FT4, with higher UPC associated with lower serum FT4 (β = − 0.29, 95% CI -0.52 to − 0.07, *p* = 0.01) (Table 2). The addition of smoking to the multivariable regression analysis did not change the association. The regression coefficient (β) of − 0.29 suggests that for every 10% increase in UPC, there will be a 0.03 pmol/l decrease in serum FT4.

**Table 2:** Regression analyses demonstrating the relationships between urinary perchlorate concentration and other potential confounding variables on serum FT4 and TSH (n = 308)

	β	SE	95% CI for β	t	p
Factors associated with serum FT4					
Urinary perchlorate^a^	−0.291	0.114	(− 0.515, − 0.066)	−2.55	**0.01**
Urinary iodine^a^	0.156	0.124	(−0.088, 0.401)	1.27	0.21
Urinary thiocyanate^a^	0.196	0.127	(−0.053, 0.446)	1.55	0.12
TPO antibody positive	−0.345	0.413	(−1.156, 0.467)	−0.84	0.4
Factors associated with serum TSH^a^					
Urinary perchlorate^a^	0.008	0.032	(−0.056, 0.071)	0.24	0.8
Urinary iodine^a^	−0.03	0.035	(−0.10, 0.039)	−0.85	0.4
Urinary thiocyanate^a^	−0.047	0.036	(−0.117, 0.24)	−1.31	0.2
TPO antibody positive	0.248	0.117	(0.02, 0.48)	2.126	**0.03**

^a^Denotes variable transformed using natural logs (allowing β coefficients to be interpreted in terms of percentage change)

Bold values are significant result in the table

Only TPO Ab positive was an independent predictor of serum TSH (Table 2). UPC or UTC was not an independent predictor of serum TSH.

The multiple regression analysis in the subgroup of women with UIC less than 100 μg/l also showed that log UPC was borderline significantly independently associated with serum FT4, with higher UPC associated with lower serum FT4 (β = − 0.33, 95% CI -0.66 to 0.001, *p* = 0.05) (Table 3). UPC or UTC was not an independent predictor of serum TSH.

**Table 3:** Regression analyses demonstrating the relationships between urinary perchlorate concentration and other potential confounding variables on serum FT4 and TSH in a subgroup of women with UIC < 100 μg/l (*n* = 174)

	β	SE	95% CI for β	t	p
Factors associated with serum FT4					
Urinary perchlorate^a^	−0.33	0.166	(−0.655, 0.001)	−1.97	**0.05**
Urinary iodine^a^	0.153	0.308	(−0.455, 0.762)	0.50	0.6
Urinary thiocyanate^a^	0.184	0.170	(−0.151, 0.520)	1.09	0.3
TPO-Ab positive	−0.316	0.512	(−1.328, 0.70)	−0.616	0.54
Factors associated with serum TSH^a^					
Urinary perchlorate^a^	0.04	0.043	(−0.046, 0.125)	0.91	0.4
Urinary iodine^a^	−0.06	0.081	(−0.214, 0.104)	−0.68	0.5
Urinary thiocyanate^a^	−0.044	0.044	(−0.131, 0.044)	−0.98	0.3
TPO-Ab positive	0.257	0.134	(−0.01, 0.52)	1.92	0.06

^a^Denotes variable transformed using natural logs (allowing â coefficients to be interpreted in terms of percentage change)

Bold value is significant result in the table

### Effect of maternal perchlorate and thiocyanate exposure on offspring birthweight

There was no correlation between maternal UPC and offspring birthweight (*r* = − 0.07, *p* = 0.2 for the whole cohort; *r* = − 0.04, *p* = 0.6 for the subgroup of women with UIC less than 100 μg/l) or between maternal UTC and infant birthweight (*r* = 0, *p* = 1.0 for the whole cohort; *r* = 0.03, *p* = 0.7 for the subgroup of women with UIC less than 100 μg/l). Subgroup analyses using birthweight (corrected for gestational age) of male and female infants separately also failed to show association between offspring birthweight and maternal UPC (*r* = − 0.09, *p* = 0.3) or maternal UTC (*r* = 0.09, p = 0.3).

## Discussion

Our study has shown that environmental exposure to perchlorate and thiocyanate is ubiquitous and the exposure to perchlorate is associated with lower circulating FT4 levels in third trimester pregnant women. We did not identify an association between exposure to thiocyanate in pregnancy and maternal thyroid hormone. Maternal exposure of perchlorate or thiocyanate was not associated with offspring’s birthweight.

Previous studies examining association between perchlorate exposure and maternal thyroid function in pregnancy have shown inconsistent results (Table 4). Although some studies [6–8, 12] found no association between UPC and maternal thyroid function, others have shown that UPC is associated with low maternal FT4 and/or high TSH [9–11]. Possible reasons for the discrepant results include diverse study populations, different iodine status, and variable gestational age at the time of perchlorate and thyroid function assessment. In our study, there was a 0.03 pmol/L decrease in maternal serum FT4 level with every 10% increase in UPC, providing additional evidence to support the association between perchlorate exposure and reduced maternal thyroid hormone levels. This is in keeping with observations outside pregnancy, where perchlorate exposure has been shown to be associated with decreased FT4 in the general population, including non-pregnant women and adolescent girls [16, 17].

**Table 4:** Studies assessing the association between urinary perchlorate concentration and maternal thyroid status in pregnancy

Author (year)	Country	N	Gestation	Median UIC (μg/l)	Median UPC (range) (μg/l)	Association of UPC with	
						Maternal TSH	Maternal FT4
Pearce, 2010 [6]	Italy	787^a^	1st trimester	50–55	5 (0.04–168)	No	No
	Wales	854^b^	1st trimester	98–117	2 (0.02–368)	No	No
Pearce, 2011 [7]	USA	134	1st trimester	144	7.8 (0.4–284)	No	No
	Argentina	107	1st trimester	130	13.5 (1.1–676)	No	No
Pearce, 2012 [8]	Greece	134	1st trimester	120	4.1 (0.2–118.5)	No	Yes (negative)^c^
Charatcharoenwitthaya, 2014 [9]	Thailand	200	1st trimester	153.5	1.9 (0.1–35.5)	Yes (positive)	Yes (negative)
Horton, 2015 [10]	USA	284	1st trimester	235 (mean)^d^	3.54 (mean)^d^	Yes (positive)	No
Steinmaus, 2016 [11]	USA	1880	Median 7 weeks	155	6.5 (0.23–177)	Yes (positive)	TT4, FT4 (negative)
Mortensen, 2016 [12]	USA	359	3rd trimester	167	4.04 (geometric mean)	No	No
This study	England	308	3rd trimester	88	2.1 (0.3–143)	No	Yes (negative)

*FT4* free T4, *TSH* thyroid stimulating hormone, *NK* not known, *UIC* urinary iodine concentration, *UPC* urinary perchlorate concentration

^a^Includes 261 women with hypothyroidism/hypothyroxinaemia (median UIC 55 μg/l) and 526 euthyroid women (median UIC 50 μg/l)

^b^Includes 374 women with hypothyroidism/hypothyroxinaemia (median UIC 98 μg/l) and 480 euthyroid women (median UIC 117 μg/l)

^c^Negative association on univariate analysis but no longer associated after adjustments in multivariate analysis

^d^Creatinine – adjusted (μg/g creatine)

The association between perchlorate exposure during pregnancy and reduced maternal serum FT4 could potentially have important clinical implications as maternal thyroid hormone insufficiency has been shown to be associated with impaired neurocognitive development of offspring and other adverse obstetric outcomes [18, 19]. In a recent study, pregnant women with subclinical hypothyroidism or hypothyroxinaemia and UPC in the upper 10% were found to have a 3-fold increased odds of having offspring with an intelligence quotient (IQ) in the lowest 10% at age of 3 years [20]. However, UPC was not associated with maternal thyroid hormone status in this cohort. Therefore, the mechanism for the association between maternal perchlorate exposure and reduced offspring IQ in this study remains uncertain although it may be due to the effect of perchlorate exposure on fetal thyroid function [20]. Another recent study has suggested that maternal perchlorate exposure in pregnancy could also affect obstetric outcomes, with maternal perchlorate exposure associated with increased birthweight in male infants, particularly in infants born prematurely [21]. However there was no assessment of maternal thyroid function in the cohort; therefore it is uncertain whether the association seen was mediated through changes in maternal thyroid hormone levels. Our study was unable to confirm the association between maternal perchlorate exposure and offspring birthweight in the whole cohort as well as in the subgroup of male infants. This is in keeping with a previous study which also found no association between perchlorate exposure in pregnancy and offspring’s birthweight, birth length or gestational age at birth [22]. However, taken together, these observations highlight the need for further investigations to examine whether environmental exposure to perchlorate during pregnancy has adverse health outcomes beyond minor changes on maternal thyroid hormone levels.

In contrast to our findings on perchlorate exposure, we were unable to show an association between thiocyanate exposure and maternal thyroid hormone levels. This may be because thiocyanate is a much weaker inhibitor of sodium-iodine symporter as compared to perchlorate [5]. However, a small number of previous studies have suggested potential effects of thiocyanate exposure on maternal thyroid status [6, 8, 9] **(**Table 5**)**. Furthermore, it is also possible that co-exposure of thiocyanate with other environmental pollutants have more deleterious impact on maternal thyroid function than exposure to an individual pollutant [10].

**Table 5:** Studies assessing the association between urinary thiocyanate concentration and maternal thyroid status in pregnancy

Author (year)	Country	N	Gestation	Median UIC (μg/l)	Median UTC (range) (μg/l)	Association of UTC with	
						Maternal TSH	Maternal FT4
Pearce, 2010 [6]	Italy	787^a^	1st trimester	55–60	373 (132–6650)	No	Yes (negative)^c^
	Wales	854^b^		98–117	471 (34–3100)	No	No
Pearce, 2012 [8]	Greece	134	1st trimester	120	413 (0.6–1635)	Yes (positive)^d^	No
Charatcharoenwitthaya, 2014 [9]	Thailand	200	1 s trimester	153.5	510.5 (68–3525)	Yes (positive)	Yes (negative)
Horton, 2015 [10]	USA	284	1st trimester	235 (mean)^e^	1006 (mean)^e^	No	No
This study	England	308	3rd trimester	88	436 (29–3290)	No	No

*FT4* free T4, *TSH* thyroid stimulating hormone, *NK* not known, *UIC* urinary iodine concentration, *UTC* urinary thiocyanate concentration

^a^Includes 261 women with hypothyroidism/hypothyroxinaemia (median UIC 55 μg/l) and 526 euthyroid women (median UIC 50 μg/l)

^b^Includes 374 women with hypothyroidism/hypothyroxinaemia (median UIC 98 μg/l) and 480 euthyroid women (median UIC 117 μg/l)

^c^Negative correlation in the euthyroid cohort on univariate analysis

^d^Positive association on univariate analysis but no longer associated after adjustments in multivariate analysis

^e^Creatinine – adjusted (μg/g creatine)

We acknowledge several limitations of the study: urinary iodine, perchlorate and thiocyanate concentrations were measured on a single spot urine sample, and thyroid hormone assessments were carried out only once. Our cohort was predominantly Caucasian, from South West England, thereby limiting generalisability.

## Conclusions

This study provides further evidence that environmental perchlorate exposure is associated lower circulating serum FT4 levels in pregnant women. This may have an adverse impact on neurocognitive development of the fetus and other pregnancy outcomes.

## Abbreviations

BMI: Body mass index
CI: Confidence interval
FT4: Free thyroxine
IQ: Intelligence quotient
IQR: Inter-quartile range
RD&E: Royal Devon & Exeter hospital
SD: Standard deviation
TPO-Ab: Thyroid peroxidase antibodies
TSH: Thyroid stimulating hormone
UIC: Urinary iodine concentration
UPC: Urinary perchlorate concentration
UTC: Urinary thiocyanate concentration
